# A half-century of research on tuberculosis: Successes and challenges

**DOI:** 10.1084/jem.20230859

**Published:** 2023-08-08

**Authors:** Barry R. Bloom

**Affiliations:** 1 Immunology and Infectious Diseases, Harvard T.H. Chan School of Public Health, Boston, MA, USA

## Abstract

Great progress has been made over the past half-century, but TB remains a formidable global health problem, particularly in low- and middle-income countries. Understanding the mechanisms of pathogenesis and necessary and sufficient conditions for protection are critical. The need for inexpensive and sensitive point-of-care diagnostic tests for earlier detection of infection and disease, shorter and less-toxic drug regimens for drug-sensitive and -resistant TB, and a more effective vaccine than BCG is immense. New and better tools, greater support for international research, collaborations, and training will be required to dramatically reduce the burden of this devastating disease which still kills 1.6 million people annually.

## Teaser

Great progress has been made over the past half-century, but TB remains a formidable global health problem. The Perspective summarizes much that has been learned and identifies key areas for which new knowledge is critically needed.

## Introduction

*There* *is a dread disease which so prepares its victim, as it were, for death; which so refines it of its grosser aspect, and throws around familiar looks unearthly indications of the coming change—a dread disease, in which the struggle between soul and body is so gradual, quiet, and solemn, and the result so sure, that day by day, and grain by grain, the mortal part wastes and withers away, so that the spirit grows light and sanguine with its lightening load, and, feeling immortality at hand, deems it but a new term* (Nicholas Nickleby, 1838, Chapter 49). So wrote Charles Dickens to describe the infectious disease that historically has killed more people than any other.

It was a privilege to be invited to present a keynote address at the Keystone TB Symposium on the 50th anniversary of the Keystone Symposia. This Perspective summarizes and expands on that presentation and reviews many breakthroughs in TB research over the past half-century and beyond that have set the stage for current and future research. It is my hope that sharing the perspective of a long-time TB researcher may provide a helpful background to a younger generation of students and researchers in the field.

Evidence from mummies in Syria and Egypt reveals that tuberculosis (TB) existed as early as 8,000 BC (Baker et al., 2015; Anastasiou and Mitchell, 2013). In the classic first-ever published study of epidemiology, John Graunt surveyed the causes of death in 1632 London using church records and reported that consumption was the second greatest cause of mortality (Graunt, 1975). Robert Koch isolated the pathogen *Mycobacterium tuberculosis* (*Mtb*) in 1882 and formulated Koch’s postulates, criteria for establishing any infectious agent as causative of disease (Koch, 1982). In 2021, *Mtb* was estimated to be responsible for 10.6 million new cases of TB and 1.6 million deaths (WHO, 2022a). It is an almost perfect pathogen, causing disease in only 5–10% and death in 1/100,000 of people infected, maintaining a large susceptible population to assure its survival. And TB persists. If one compares public investments in research just for COVID-19 RNA vaccines ($31.9 billion; Lalani et al., 2023) versus all of TB research for 2022 ($1 billion; TAG, 2022), it is clear that lack of funding has been a major impediment to developing new tools to combat TB.

## Context

### A structural problem

**Figure 1.**
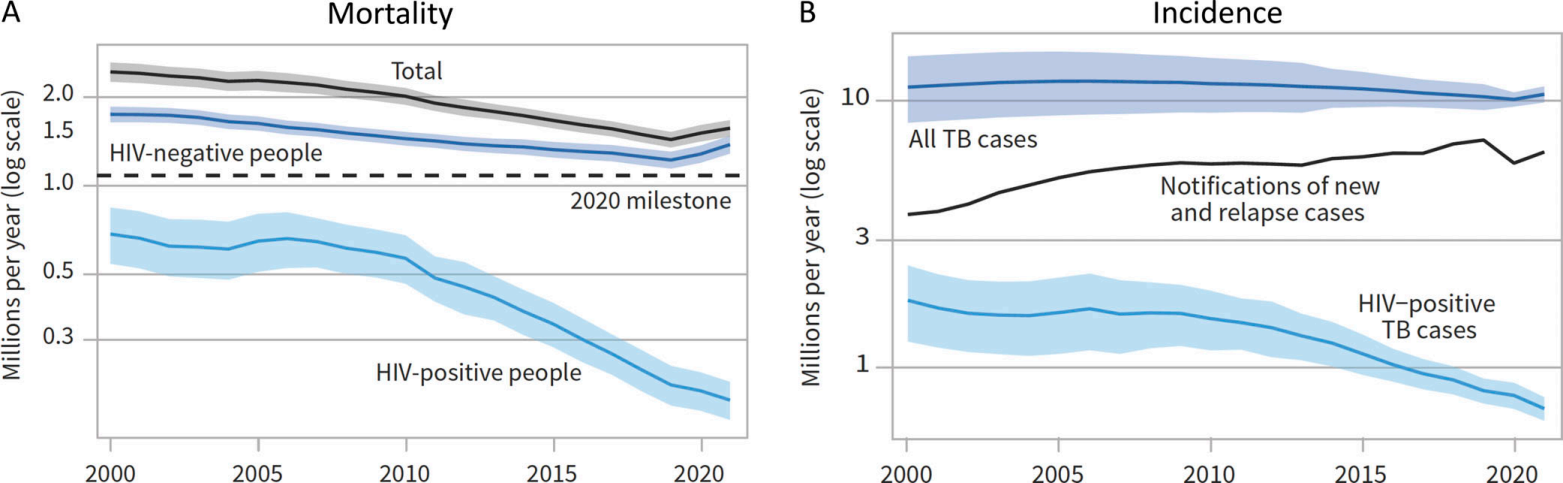
**Global trends in the estimated number of TB deaths and mortality rate, 2000–2020.** Shaded areas represent the uncertainty interval. The horizontal dashed line shows the 2020 milestone of the WHO TB strategy. This image is reprinted from the WHO Global Tuberculosis Report 2022 (WHO, 2022a).

If we consider the current epidemiological picture, the good news is that mortality from TB is declining (Fig. 1 A), but the reduction in deaths is mainly attributable to antiretroviral treatment of patients coinfected with TB and HIV. Tragically, the incidence of TB hasn't changed in decades (Fig. 1 B). The overarching finding of the World Health Organization (WHO) report from 2022 is that the COVID-19 pandemic has had a damaging impact on access to TB diagnosis, treatment, and the burden of TB disease, and that there has been a large global fall in the number of people newly diagnosed with TB and reported (i.e., officially notified) in 2020 compared with 2019 (WHO, 2022a; Fig. 1 C). For TB to persist unchanged in incidence around the world is an indictment of the paucity of funding for research, non-optimal strategy and infrastructure for TB, and of the limited engagement of the scientific community.

### An implementation problem

In groundbreaking work on the cascade of care in India, an analysis revealed that only about 60% of the total estimated prevalent cases of TB were diagnosed and fewer than 50% completed treatment and were cured in 1 yr (Subbaraman et al., 2019). The program to eliminate TB as a global public health problem has long been based on the premise that if people are sick, they will come to a healthcare facility, they will be diagnosed, they will receive proper treatment, and they will be cured. However, the data indicate that this assumption is wrong at every level (Subbaraman et al., 2019). TB is not like malaria, where 3 d of treatment in children is sufficient to ameliorate the disease. Treatment for TB is complex; multiple drugs are taken over many months. The emerging problem of drug resistance is continuing. Even though treatment regimens have been shortened and improved, getting treatments to people who need them, maintaining them, and monitoring their efficacy is really challenging. If the fundamental goal in controlling any infectious disease is to interrupt transmission, we are failing. And that should represent an urgent call for better tools—for more effective diagnosis, treatment, prevention, and implementation of TB control.

## Transmission

The classic picture of aerosol transmission is a backlit photograph showing visible droplets from a patient coughing. But the visible droplets seen are not the aerosol nuclei thought to transmit *Mtb* to the lower lung, which are 100–1,000 times smaller. This represents a useful point for advocacy for TB research—namely, that the greatest risk for *acquiring* tuberculosis is breathing.

A recent breakthrough by Wood and colleagues has enabled the measurement of total and viable tubercle bacilli in aerosols of patients with TB. They compared the amount of *Mtb* released over the course of an hour and compared the amounts from coughs and ordinary tidal breathing (Dinkele et al., 2022). Despite the fact that more bacilli are released per cough, just breathing over the course of 24 h can account for 90% of *Mtb* released. As pointed out in Scriba et al. (2021), the clear implication is that TB can be transmitted from asymptomatic individuals, not merely from the small number of symptomatic cases that show up at a clinic. This may explain why the incidence curve has not changed in 30 yr. As Wood’s research shows (Dinkele et al., 2022), the major risk for *transmitting* TB is breathing.

A fundamental problem with the global TB elimination strategy is that it has been almost solely based on passive case finding, which means waiting for people to become sick enough to seek medical care. In a comprehensive survey of diagnosed TB, about 50% of infections, a massive number, were found to be subclinical (Frascella et al., 2021) A very exciting recent finding from Fox, Marks, and colleagues in Australia revealed greater effectiveness of active versus passive case finding (Fox et al., 2018; Marks et al., 2022). They examined 25,000 household contacts of TB patients in Vietnam. They were able to find two and a half times more sputum-positive cases of TB than they would have had only the cases seen in clinic been followed. They were thus able to lower the community rate of infection by 22% in 3 yr by introducing earlier treatment regimens. As Marks et al. (2022)’s data suggest, there are lots of people in the community who feel well but have asymptomatic or chronic TB, which can subtly progress to active disease. Unless provided with preventative therapy, they represent an additional source of people who contribute to transmitting infection over time.

That the WHO had for years not prioritized active case finding, or more recently recommended it only for geographically defined subpopulations with a high level of undetected TB (0.5% prevalence or higher) and vulnerable or marginalized groups, is clearly understandable because it is more expensive in terms of the personnel required and costs (WHO, 2021). Active case finding requires gaining access to households to test contacts once the TB index case has been diagnosed; and improved, simpler diagnostics; and delivering preventive treatment to prevent progression. This requires a good healthcare system and is expensive. One way to make it less expensive, of course, would be to have inexpensive but sensitive point-of-care diagnostic tests.

## Structure of *Mtb*

The cell wall of the bacillus serves as a major virulence determinant of the pathogen and contains the target of many drugs. One of the great achievements in TB research has been the delineation of the beautiful but complex structure of the cell wall (Brennan, 2003; Brennan and Besra, 1997; Dulberger et al., 2020; Niederweis et al., 2010). Along with structure are the complex lipid synthetic pathways, including mycolic acids and virulence determinants (e.g., phthiocerol dimycocerosates), and secretion systems (e.g., Esx1), responsible for disrupting cell membranes and enabling penetration into cells and cytoplasm. Phthiocerol dimycocerosate was shown to be necessary for *Mtb* virulence (Cox et al., 1999). Some lipids scavenge antimicrobial radicals generated by immune responses (Chan et al., 1989), and other lipids prevent the penetration of many drugs (Nasiri et al., 2017). There are specialized import and export pumps that render the pathogen resistant to multiple drugs (Abrahams and Besra, 2021). Many new drugs being developed for TB are designed to target the three major cell wall layers, mycolic acid, arabinogalactan, and peptidoglycan (see reviews by Dartois and Rubin, 2022; Edwards and Field, 2022; Xu et al., 2022).

## Pathogenesis

**Figure 2.**
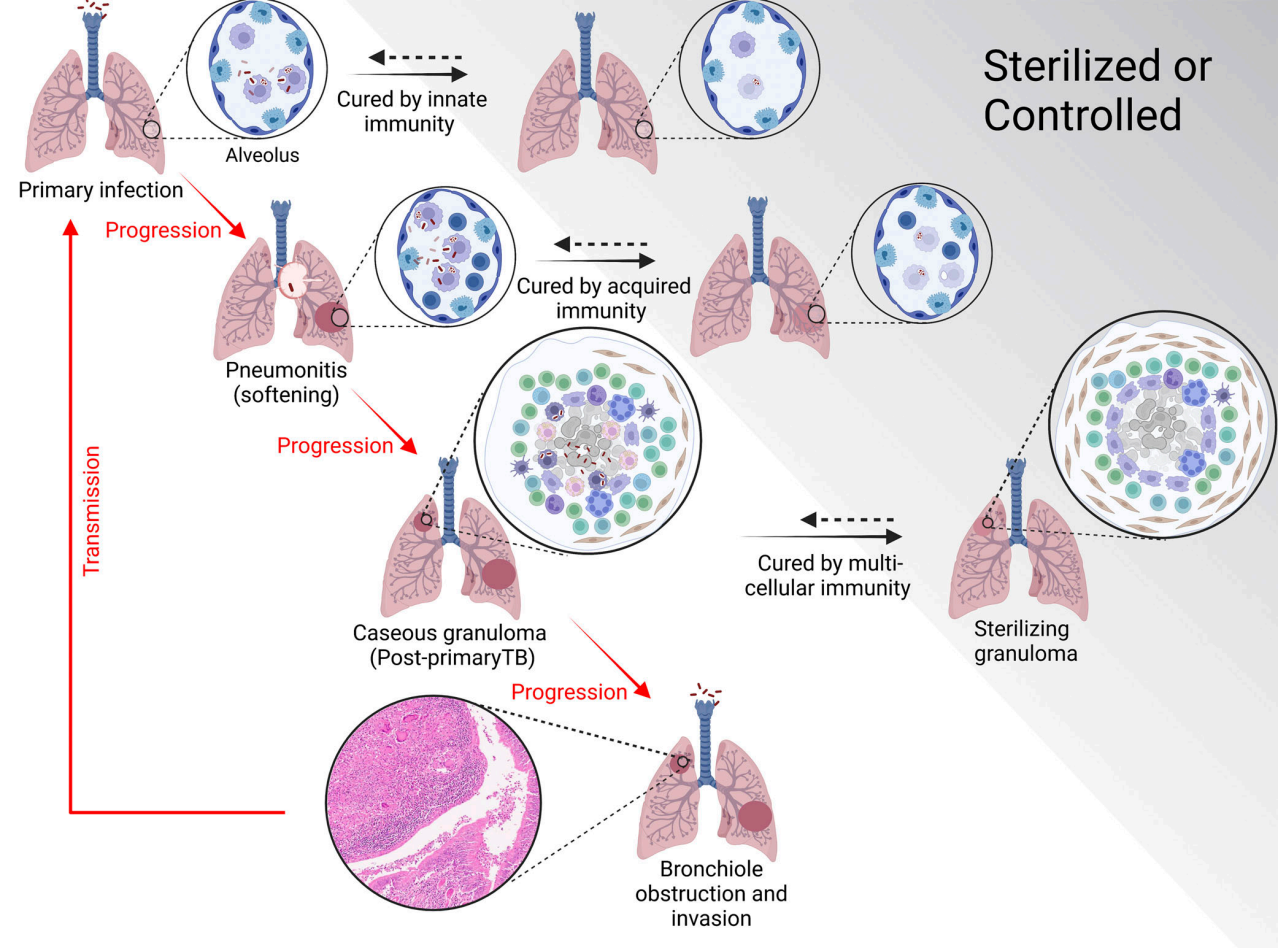
***Mtb* pathway to pathogenesis schematic diagram.** The vertical arrows indicate the pathway of disease progression. The horizontal arrows indicate stages at which the pathogen can be controlled or sterilized. The dotted arrows indicate that each state of control can be reversed (Modlin, R.L. and B.R. Bloom, unpublished figure).

It is astonishing how exquisitely and accurately the giants in the history of TB research described the origin and progression of the pathogenic process of pulmonary TB disease (Rich, 1951; Canetti, 1955; Medlar, 1955; Hunter, 2020). Infected macrophages from the alveolus can stimulate local lymph nodes to confront the pathogen. If that fails, infected TB bacilli or infected macrophages can migrate through the lymphatics or blood (Verma et al., 2020), ultimately to the more oxygen-rich upper lung, and progress to form a granuloma that focuses and organizes the host’s multifaceted immune response effort to wall off and kill the bacilli. If the bacilli are not killed, the disease process can progress ultimately to obstruction of a bronchus and coughing to release bacilli (Fig. 2). Each stage is potentially reversible. While extrapulmonary lesions occur in lymph nodes and other organs, they are not a major contributor to transmission. The current focus of research is almost exclusively on the organized lung granuloma.

Granulomas are the organized accumulation of multiple cell types representing the body’s final stand to prevent invasion of bronchioles and ultimately dissemination (reviewed in Cohen et al., 2022; Pagán and Ramakrishnan, 2018). Recent publications have provided in-depth findings on the cellular components of the granuloma both in non-human primates (NHP; Ford et al., 2011; Lin et al., 2006, 2014, 2016; Gideon et al., 2015, 2022) and human TB (Lenaerts et al., 2015; Marakalala et al., 2016; Hunter, 2020; McCaffrey et al., 2022) and in leprosy granulomas (Ma et al., 2021). It is important to recognize that the infection can be interrupted and the bacilli killed or controlled by innate or acquired cellular immune responses at every stage of disease. This is strongly supported by findings of remnants of the TB bacillus isolated from healed TB lesions that have been found in autopsies of many people who died of other causes (Medlar, 1955). The progression from infected alveolus to granulomas and transmission reflects a failure of the immune responses at each stage.

## Two puzzles of host responses

A fundamental question to ask is how it is possible, in the same lungs, to have TB granulomas which (i) are sterilized; (ii) control growth of *Mtb*; and (iii) allow *Mtb* to grow to kill the host? The answer was probably found by Robert Koch, who showed that intradermal inoculation of *Mtb* into naïve guinea pigs led to progressing lesions that ultimately killed the animal. In contrast, in similarly infected animals, inoculation of *Mtb* in a distant site led to small indurated lesions that necrosed and then healed with scaring, although the animals ultimately died from the primary lesion (Koch, 1982). This was defined as the Koch phenomenon. When Koch injected tuberculin, an extract of *Mtb*, as a vaccine in humans who had TB, the necrotic phenomenon in the lungs killed some of them, leading to Koch's self-imposed exile for many years (Bloom and Murray, 1992; Kaufmann, 2021). The principle is general and seen in other circumstances, for example, during the reintroduction of schistosomes (Clegg et al., 1971) or cancer cells (Gershon et al., 1967) in animals, where the primary inoculation progresses but secondary lesions are resolved. The current terminology is “concomitant immunity.” In TB, we would hypothesize that the first bacillus to grow into a lesion or granuloma gets a head start but generates concomitant immunity to enable secondary lesions either to be contained or sterilized. The variations in granulomas seen in NHP and *Mtb* challenge experiments, which show that primary infection significantly reduced the growth and increased the killing of barcoded *Mtb* in the rechallenge, confirm the concomitant immunity hypothesis (Cadena et al., 2018).

Several historical breakthroughs in host responses were the discovery of cytokines, non-antibody proteins produced by lymphocytes, which derived from the demonstration that secreted proteins of tuberculin-sensitive lymphocytes stimulated by purified protein derivative (PPD) in vitro activated macrophages (Bloom and Bennett, 1966; David, 1966) and the finding that there were functionally distinct T cell subsets in mice, Th1 and Th2 (Mosmann et al., 1986), that produced different cytokines. These subsets were shown to exist in human leprosy lesions, produced distinct cytokine patterns, and were predictive of the different disease outcomes in human leprosy (Yamamura et al., 1991; Salgame et al., 1991). A major focus of current research is to analyze the dynamics of multiple cell subsets in pulmonary TB lesions using sophisticated technologies, for example, using immunohistological staining and micro-CT of human lesions (Wells et al., 2021). Single-cell RNA sequencing (RNAseq) has identified the transcriptional profiles and localization of the multiple cell types found in organized NHP granulomas (Gideon et al., 2022)—and there are many: Th17 T cells, macrophage subsets and dendritic cells, natural killer (NK) cells, neutrophils, eosinophils, B cells, mast cells, endothelial cells, and fibroblasts (Ma et al., 2021; Marakalala et al., 2016; Dheda et al., 2019; McCaffrey et al., 2022; and reviewed in Cohen et al., 2022; Pagán and Ramakrishnan, 2018). Spatial mRNAseq has enabled tissue localization of individual cell subtypes in leprosy granulomas, revealing multiple different cell types expressed mRNAs of putatively antimicrobial genes (Ma et al., 2021). At later stages, some of these cell types mobilizing in granulomas that may be important in controlling the infection may also contribute to greater pathology and damage to the lung and other infected tissues. Regrettably, much less is understood about early lesions in TB (Hunter, 2020), in which protection can occur without significant tissue and lung damage. Together, these findings suggest simply that it may “take a village” to protect against pathogenic mycobacteria.

Another of the mysteries of TB pathogenesis has been what triggers the characteristic cough of TB. It was known for half a century (Middlebrook, 1950) that *Mtb* sulfolipids were associated with virulent *Mtb* strains but did not appear to affect either growth or lesions in animals. One potential mechanism has recently been reported, namely that *Mtb* sulfolipid SL1 can trigger nociceptive neurons involved in the characteristic cough reflex (Ruhl et al., 2020). Triggering a cough can lead to the rupture of a bronchiolar block and release aerosols, thus reinitiating the cycle of transmission.

## Creating a genetic system in mycobacteria

It is now not possible to understand the scientific basis of microbial pathogenesis of any infectious disease, drug, or vaccine without a genetic system that reveals the whole genome sequence and enables the introduction and deletion of genes. *Mtb*, which is a BSL-3 biohazard, grows slowly, lacks natural plasmids, has a waxy cell wall prone to clumping, and was notorious for many years for being refractory to genetic transformation or manipulation. To overcome these hurdles, Jacobs et al. (1987) were able to create a genetic system in mycobacteria using phages capable of infecting *Mycobacterium smegmatis*, a rapidly growing, nonpathogenic mycobacterium, an unsung hero in TB research now widely used as a uniquely useful model organism for study (Sparks et al., 2023). To manipulate genes and enable their transfer to *Mtb*, recombinant shuttle phasmids were constructed, which are chimeras containing mycobacteriophage DNA into which an *Escherichia* *coli* cosmid is inserted. They can be manipulated in *E. coli* as plasmids and infect mycobacteria as phages, and transfer DNA across both genera. The shuttle vectors permitted the introduction of foreign DNA in mycobacteria and the expression and deletion of genes in mycobacteria for the first time (Snapper et al., 1988). It was then possible to stably integrate foreign genes into the *attB* site of the mycobacterial genome and express foreign antigens (Stover et al., 1991), making the development of recombinant vaccines possible (reviewed by Kaufmann [2021] and Marinova et al. [2017]). Sassetti and Rubin introduced the *Drosophila mariner* transposon to randomly mutagenize *Mtb*, which first enabled the definition of the essential genes for growth and pathogenesis in mice (Sassetti et al., 2003; Joshi et al., 2006). The heroic effort of Cole and colleagues to delineate the complete DNA sequence of *Mtb* has provided the map for essentially all future genetic analysis of the pathogen (Cole et al., 1998).

What has the genetic system in *Mtb* enabled researchers in the field to do? It is the basis for compiling genomic databases of mycobacterial isolates from all over the world, which in turn provides the basis for studying variations and evolution of strains. With that, Gagneux and colleagues identified seven distinct phylogenetic lineages of *Mtb* that likely co-evolved with the great human migrations from Africa over millennia (Comas et al., 2013).

The complete DNA sequence of *Mtb* enabled molecular identification of *Mtb* in clinical sputum samples and detection of drug resistance in 90 min, e.g., Xpert MTB/RIF assay, a nucleic acid amplification test that uses a disposable cartridge, obviating the need for long culture times (Boehme et al., 2010). The DNA sequence of *Mtb* also enabled the development of several molecular diagnostic assays that can rapidly distinguish infection by *Mtb* infection from bacille Calmette–Guérin (BCG) immunization (WHO, 2022a, WHO, 2022b). Kaufmann et al. introduced genes from a different bacterial pathogen, listeria, into the BCG vaccine that enabled it to survive longer in phagosomes and generate more cytotoxic T lymphocytes in animal models, reviewed in Kaufmann (2021). The DNA sequence of *Mtb* has additionally enabled the deletion of genes important for virulence in *Mtb* that Martin and colleagues used to create a live attenuated *Mtb* vaccine candidate now in clinical trials (Martín et al., 2021).

Also derived from knowledge of the *Mtb* genome, and quite unanticipated, was the discovery by Hatfull, Jacobs, and colleagues that a phage from the Bronx Zoo that made large plaques (BxB1, for “Bronx Bomber”; Nkrumah et al., 2006), and which possesses a highly accurate and efficient integrase for site-specific integration of genes in mammalian cells, is now widely used in human and animal models of gene therapy (Hosur et al., 2022).

## Mechanisms of protection and pathogenesis against TB determined using animal models

No animal model for TB faithfully reproduces the precise characteristics of TB in humans. Studies of the mechanisms and dynamics of protection and pathogenesis in human TB are difficult, particularly changes occurring over time in the lungs and lymph nodes of living patients. Several models, however, have provided specific insights into mechanisms of protection and pathogenesis that may be occurring in human TB patients.

The mouse model is the most comprehensively studied for the immune responses to infection since reagents detecting immune molecules are widely available, as are genetically modified knockout mice. The lung pathology of active TB disease is very different in most mouse strains than that observed in human TB, showing many diffuse lesions without classical granuloma structure. Most mouse inbred strains are relatively resistant to *Mtb* infection; for example, the widely used C57Bl/6 mouse, on which background most knockouts are made. The ability to knockout genes of the immune response showed that both MHC Class II–reactive CD4 T cells and MHC Class I–restricted CD8^+^ T cells were important in protection against TB in mice (Flynn et al., 1992) and in macaques (Chen et al., 2009; Lin et al., 2012). Moreover, cytokines including IFN-γ and TNFα (Flynn et al., 1993, 1995; Cooper and Flynn, 1995; Flynn and Chan, 2001), IL-12 (Cooper, 2009; Cooper et al., 1997), and IL-1 (Mayer-Barber et al., 2014) were all involved in protection against *Mtb* infection. IL-12, IFN-γ, and signaling molecules of the Th1 axis (Gros and Casanova, 2022), as well as TNF (Keane et al., 2001), have all been shown to be associated with protection against mycobacterial infections in humans as discussed in more detail below.

There has long been a question of whether there are differences in host genetics that could explain why some humans exposed to *Mtb* infection develop disease while others do not. The susceptible strain C3Heb/FeJ was the first mouse model to exhibit lung pathology and caseous granulomas similar to those observed in human TB (Kramnik et al., 2000; reviewed in Kramnik, 2008). They developed a congenic mouse strain by classical breeding between the susceptible and resistant strains and identified a locus that was associated with susceptibility to TB (Pan et al., 2005). That locus (sst^s^) was later shown to encode a transcription factor, SP140, which bestowed enhanced resistance in mice by suppressing the type I IFN response (Ji et al., 2019). Inbred congenic populations of mice derived from random crosses of wild mice and basic strains (Collaborative Cross) have been shown to exhibit a wide range of genetic diversity and highly variable immune responses to the *Mtb* challenge and BCG vaccination (Smith et al., 2022). How the variable responses involved in these mouse-congenic strains relate to human susceptibility remains to be determined.

The NHP model is widely assumed to exhibit responses and pathology closest to human disease (Lin et al., 2006). There are two species of NHP that present different courses of disease after challenge: rapid and acute, resembling primary progressive human TB (rhesus macaques), or chronic and latent (Pena and Ho, 2016; cynomologous macaques). As in mice, both CD4 (Lin et al., 2012) and CD8 T cells (Chen et al., 2009) are required for the control of infection. Using barcoded *Mtb*, Fortune, Flynn, and colleagues established that each lesion could be caused by a single bacillus (Martin et al., 2017). In a significant advance, positron emission tomography–computed tomography (PET-CT) scanning enabled the study of the dynamics of lesion progression in vivo and the impact of drugs and vaccines (Chen et al., 2014). Older studies in NHP found that intravenous and aerosol immunization with BCG were comparably protective (Barclay et al., 1970; Anacker et al., 1972; Darrah et al., 2020) against *Mtb* infection. Importantly, Darrah et al. (2020) using PET-CT scanning found that intravenous BCG immunization was strikingly more protective against *Mtb* infection than aerosol immunization, resulting in sterilizing immunity.

Infection of zebrafish larvae with *Mycobacterium marinum*, which grows at 32°C, has facilitated the study of innate immunity as a model of human TB and allows visualization of the development of lesions following granuloma formation in vivo (Pagán and Ramakrishnan, 2018). The model has also led to defining the role of different virulence factors in different steps of infection (Davis et al., 2002), providing a platform for rapid in vivo anti-mycobacterial drug screening (Takaki et al., 2012). However, this model has limitations: zebrafish have gills, not lungs, and larvae, which have innate responses, lack lymphocytes, and thus lack the adaptive immune responses shown to be fundamentally important in the protective response in human TB.

A model worthy of mention is the mini-pig, which is almost totally resistant to death from TB infection and exhibits organized granulomas encapsulated by many layers of fibroblasts that effectively wall off the pathogen (Gil et al., 2010). Other models—e.g., the guinea pig, which is so susceptible that statistically one tubercle bacillus will kill it; rabbits, which are a model for TB meningitis; and the bovine model—have niche advantages but are less well-studied will not be reviewed here.

Few models have provided greater insight into the pathogenesis of granulomas than studies of human leprosy caused by *Mycobacterium leprae*. Understanding leprosy’s clinical and immunological spectrum has provided the basis for recognizing the TB disease spectrum. Leprosy presents a clinical and immunological spectrum in which clinical manifestations correlate with the type of immune response (Ridley, 1974). At one pole, the localized form of leprosy, tuberculoid leprosy, skin granulomas contain macrophages, T cells, and few bacilli and are self-limited, indicating that the immune response efficiently contains the infection. At the opposite end of the spectrum, lepromatous leprosy, the disseminated form of the disease, has many lesions containing acid-fast bacilli within macrophages, which are unable to kill them. The comparable spectrum analogy in TB would be sterilized or controlled TB infection at one pole, and latent TB, chronic TB and active TB along the spectrum, and miliary disseminated TB at the other pole. Because leprosy lesions occur only in the skin, the dynamics of the disease are easily accessible to study in contrast to pulmonary TB. It is the first human disease in which T cell subsets were demonstrated in lesions and found to predict the outcomes of disease. T cell clones from healing lesions produced IFN-γ, IL-2, and TNFα, and those in disseminating lepromatous lesions produced IL-4 and IL-10 (Yamamura et al., 1991; Salgame et al., 1991). Borderline leprosy exhibits elements of both and can undergo shifts toward either pole in reactional states. There is another example in which leprosy showed the way to a fundamental understanding of tuberculosis. The first analysis of the genome of any mycobacterial species was not of the genome of *Mtb* but of *M. leprae*. When a contig map was largely complete for *M. leprae*, the organization of DNA fragments of *Mtb* was facilitated greatly by comparing them with those already known from *M. leprae* (Philipp et al., 1996). It was this analysis that enabled Cole and his intrepid collaborators to successfully complete the organization and sequencing of the *Mtb* genome (Cole et al., 1998).

## Human responses to *Mtb* infection

TB represents a clinical and immunological spectrum ranging from a group of repeatedly exposed individuals who exhibit no *Mtb* skin test (TST) positivity or IFN-γ production by peripheral T cells to disseminated disease due to the lymphatic (Hunter, 2020) or hematogenous (Drain et al., 2018) spread of tubercle bacilli to the lungs and other organs. There is wide variation in studies on the time between infection with *Mtb* and progression to active disease, commonly referred to as latent TB (Behr et al., 2021). Epidemiologic evidence for the persistence of *Mtb* includes the bimodal age distribution of new cases in China, the first peak in young people representing recent transmission, and the second in people >65 yr interpreted as reactivation (Harris et al., 2019). In the US, two-thirds of men developing TB are >65 yr, consistent with late reactivation (CDC, 2020). In addition, activation of TB occurs in a portion of healthy tuberculin-positive individuals by HIV (Pawlowski et al., 2012) or anti-TNF therapy (Keane et al., 2001). However, some recent analyses suggest that the majority of *Mtb*-infected individuals who progress to active TB disease will do so within months or within 5 yr (Sutherland et al., 1982; Behr et al., 2018, 2019), although there remain others whose reactivation occurs over years (Wiker et al., 2010).

The spectrum of latent tuberculosis in humans is likely represented by a broad range of responses that occur following infection with *Mtb* (Barry et al., 2009; Pai, 2010). These responses support the formation of physiologically distinct granulomatous lesions that provide microenvironments that may differ in their ability to control or support mycobacterial growth as has been investigated in experimental models. Asymptomatic and chronic TB (Marks et al., 2022) with no or minimal symptoms, but likely trending to progression to release of viable *Mtb*, may transmit infection and create local outbreaks (Mindra et al., 2017). The importance of effective CD4^+^ T cell responses in protecting against TB has nowhere been more strikingly demonstrated than by the susceptibility of HIV-infected individuals to *Mtb* infection and disease (Pawlowski et al., 2012). Prior to anti-HIV drug treatment, TB was the largest attributable cause of death in HIV-infected individuals (Gupta et al., 2015, WHO, 2022a).

Many studies have sought to characterize critical human mechanisms involved in protection against TB (reviewed in Pai, 2010; Cliff et al., 2015; Modlin and Bloom 2013; Bloom and Modlin, 2016). Cytokines such as IFN-γ, TNFα, IL-1, and IL-23 show correlations with resistance to disease (reviewed in O’Garra et al., 2013). In vitro studies have indicated that CD4 T cells, CD8 T cells, Th17 cells, and dendritic cells are involved, and that macrophages can be activated in vitro to kill or inhibit the growth of *Mtb*. However, the requirements for these immune molecules in protection were demonstrated in animal models, as described above, and in key genetic studies on Mendelian susceptibility of humans to mycobacterial disease, characterized by susceptibility to weakly virulent mycobacteria (i.e., live BCG vaccines and environmental mycobacteria) in otherwise healthy patients (Casanova and Abel, 2002). Multiple gene variants associated with the inability to resist infections by other mycobacterial species include IFN-γ, IFN-γR1, IFN-γR2, STAT1, and IRF1 deficiencies and TYK2 (Casanova and Abel, 2002, 2022). The importance of IFN-γ in human severe TB is confirmed by monogenic deficiencies in IL12Rβ1 and IFN-γR (Abel et al., 2018). Anti-IFNγ neutralizing autoantibodies have been reported in multiple studies of disseminated non-tuberculous mycobacteria (NTM) infections (Kampmann et al., 2005). Conversely, there are highly *Mtb*-exposed individuals whose T cells fail to produce IFN-γ who nevertheless appear to be “resisters” to TB (Davies et al., 2023). The question of which cytokines are essential for resistance to infection and disease is complex and remains unresolved.

Macrophages are critical to every aspect of pathogenesis and protection. When *Mtb* is phagocytosed by human and animal macrophages activated by cytokines, bacilli can be contained or killed in phagolysosomes (Thoma-Uszynski et al., 2001). A major bacterial mechanism of pathogenesis of TB is the secretion of ESX1, a pore-forming secretion system molecule that provides access to the cytoplasm nutrients, and for *Mtb* products to enter the cytoplasm and kill the macrophages (Wong, 2017). *Mtb* also has the ability to escape from vacuoles and grow in the cytoplasm, where antimicrobial mechanisms are limited (Teitelbaum et al., 1999; van der Wel et al., 2007). If not killed, bacilli grow and kill the macrophages from which they have been shown by live-cell imaging to grow and disperse more rapidly from dead than live macrophages (Mahamed et al., 2017). In the mouse, activated macrophages kill *Mtb* primarily by the production of nitric oxide (Chan et al., 1992; MacMicking et al., 1997). While human macrophages express inducible nitric acid synthase (iNOS) in vivo (Nicholson et al., 1996; Fang and Vázquez-Torres, 2019), the levels may be insufficient in human macrophages in vitro to kill *Mtb* (Thoma-Uszynski et al., 2001). There is evidence that macrophages directly isolated from human alveolar lavage may express elevated iNOS levels (Fang and Vázquez-Torres, 2019) but whether the levels of NO are sufficient to kill *Mtb* in vivo remains unclear. At least three mechanisms have been shown to enable activated human macrophages to kill *Mtb* in vitro. One is the production of antimicrobial peptides, cathelicidin, and defensins that damage the cell wall (Liu et al., 2006). A second is the injection of the antimicrobial peptide, granulysin, by antimicrobial CD8 CTL cells, which together with perforin and granzymes, enter the macrophages and kill the bacteria (Balin et al., 2018; Stenger et al., 1998). A third is the uptake of IL-26 secreted by activated IL-17 T cells which can enter macrophages and kill the bacteria intracellularly (Dang et al., 2019). Failure to kill the *Mtb* intracellularly leads to enhanced growth and likely dissemination (Mahamed et al., 2017).

Berry and O’Garra showed that transcriptional signatures from human whole blood could distinguish individuals with no disease from those with active TB (Berry et al., 2010). The signatures revealed a strong IFN response, dominated by type I IFNs that correlated with the lung radiographic extent of disease and was diminished upon effective treatment. Type I IFNs have been shown to inhibit the activation of macrophages by IFNγ to control mycobacterial infection in vitro and to induce cytokines such as IL-10 (Teles et al., 2013; McNab et al., 2014; reviewed in O’Garra et al., 2013). In vivo studies have shown that type I IFN exacerbates TB in mice by increasing neutrophil inflammation and neutrophil extracellular trap formation (NETosis) in lungs of *Mtb-*infected mice (Moreira-Teixeira et al., 2020) and by inhibiting the protection by IL-1 against *Mtb* infection (Mayer-Barber et al., 2014; Ji et al., 2019). It is an intriguing hypothesis that type I IFNs may have evolved selectively to combat viral infections, and that type 2 IFNs (IFN-γ) function primarily to protect against intracellular pathogens (Teles et al., 2013; McNab et al., 2014).

Significant differences exist between responses in animal models and humans. For example, although oxygen radicals and H_2_O_2_ produced by mouse macrophages are able to kill most intracellular bacterial pathogens in vitro, they were not effective against *Mtb*, although nitric acid was in vitro (Chan et al., 1992), and iNOS was essential in vivo (MacMicking et al., 1997). In contrast, the killing of *Mtb* by activated human macrophages in vitro was found to be mediated primarily by antimicrobial peptides, cathelicidin, and defensins, generated through a novel IL-15 and a vitamin D–dependent pathway (Liu et al., 2006). This pathway was essential for IFN-γ induced killing by human macrophages in vitro (Fabri et al., 2011). Of particular interest, a high proportion of individuals of African descent are hypovitaminemic for vitamin D (Matsuoka et al., 1991), and Blacks have a sixfold greater incidence of TB than whites in the US (Stead et al., 1990) While their activated macrophages were poorly able to kill *Mtb* in vitro, the addition of vitamin D augmented their killing (Liu et al., 2006). However, recent clinical trials of vitamin D supplementation in people hypovitaminemic for vitamin D at risk of TB failed show a lower incidence of clinical TB (Ganmaa et al., 2017) or of cancer and cardiovascular disease (Manson et al., 2019), suggesting that there may be other factors than vitamin D levels that may be limiting.

## Diagnostics and prediction of TB

For a century, acid-fast staining of sputum smears assessed by microscopy of patients suspected of having TB has been the standard diagnostic test, but it has low sensitivity. Some adults, HIV^+^ individuals, and most children cannot produce sputum (Broger et al., 2023; Davies and Pai, 2008), and TB diagnosis in children is difficult, often requiring gastric lavage, which is not possible in high-burden TB countries (WHO, 2022a). For a more definitive diagnosis, cultures of organisms from sputum have been used, but it takes weeks for cultures to become positive and patients often fail to report back to the clinic, especially in high-burden TB countries.

The TST has been used to identify individuals who had been exposed to mycobacterial antigens. It is an intradermal challenge with PPD of *Mtb*, where induration at the skin test is assessed 24–48 later. This test only measures past or present infection and cannot distinguish between infection with *Mtb* and BCG vaccination. A major improvement has been the development of the IFN-γ release assays based on the measurement of IFN-γ released from *Mtb*-specific antigen-stimulated lymphocytes (Mazurek and Villarino, 2003). The advantages of this test are that it can distinguish exposure to *Mtb* from BCG and is easy to perform. Neither TST nor IGRA assays have high predictive value for diagnosing active TB and can only determine exposure/infection with *Mtb*, not clinical disease (Rangaka et al., 2012).

A major breakthrough was made with the development of gene amplification technology that enabled the development of automated devices, such as Gene Xpert (Boehme et al., 2010) and TruNat (Gomathi et al., 2020), which in 90 min can identify whether a clinical sample contains *Mtb* and whether it is rifampicin-resistant, indicating that it contains a drug-resistant or multidrug-resistant (MDR) strain. Other PCR tests such as the laminar flow Hain test are less expensive and provide comparable sensitivity (Nathavitharana et al., 2017). One must note that 50% of people in TB high-burden countries lack access to such molecular diagnostics (WHO, 2022a).

An exciting new technology just being applied to TB diagnosis is CRISPR-cas, which has the potential to overcome major barriers to diagnosis in low- and middle-income countries due to its cost and simplicity. As a diagnostic tool, it allows the detection of either *Mtb* RNA with cas13a (Gootenberg et al., 2018; Kellner et al., 2019) or DNA with cas12a (Chen et al., 2018) in clinical samples. Studies are currently underway to test the specificity and sensitivity of these CRISPR assays in TB, and early published findings are encouraging, indicating greater sensitivity than culture or Xpert with no loss of specificity (Ai et al., 2019; Huang et al., 2022). In addition, they can be used in the field in resource-limited settings and at very low cost (Myhrvold et al., 2018). The assays offer exciting possibilities for the improvement of TB diagnosis since they have the sensitivity to detect early infection and resistance mutations and show promise for household contact active case-finding programs.

Host blood transcriptional signatures of TB were proposed to support TB diagnosis; however, there was little consensus between different reduced blood signatures for TB risk and/or diagnosis, and there was a concern about their ability to distinguish TB from confounding diseases, particularly viral infections (reviewed in Singhania et al., 2018; Mulenga et al., 2021). The capability of lymphocyte transcriptional signatures to predict who would develop active disease was suggested in studies of IGRA-positive adolescents in South Africa (Zak et al., 2016). However, a recent randomized controlled clinical trial for biomarker-guided tuberculosis-preventive chemotherapy with isoniazid and rifapentine (3HP) in individuals with early positive signatures failed to prevent the progression to active disease and suggested that the signature was detecting incident TB (Scriba et al., 2021) and potentially also detecting intercurrent viral infection. This highlights potentially important challenges for the implementation of host transcriptional biomarkers as new tools for TB control. Subsequently, although many different signatures have been suggested to be effective predictors as well (Singhania et al., 2018; Gupta et al., 2020), it is unclear whether these signatures are indeed detecting asymptomatic disease or early infection (Tabone et al., 2021). Since the host blood transcriptional signature of TB has been reported to be diminished with effective TB treatment, it is likely that transcriptional biomarkers to monitor TB treatment would provide added value to improve clinical management of the disease, as discussed in more detail below.

For testing individuals who cannot produce sputum, two serologic lateral flow tests detecting lipoarabinomannan in urine have been recommended by the WHO for screening HIV^+^ patients for TB (Dhana et al., 2022). While of modest sensitivity, they have the advantage that for many patients who cannot produce sputum, almost all can produce urine. In addition, preliminary findings on a novel sensitive blood-based molecular diagnostic tool to allow earlier diagnoses in TB patients have recently been reported (Verma et al., 2020).

## Advances in TB treatment

**Figure 3.**
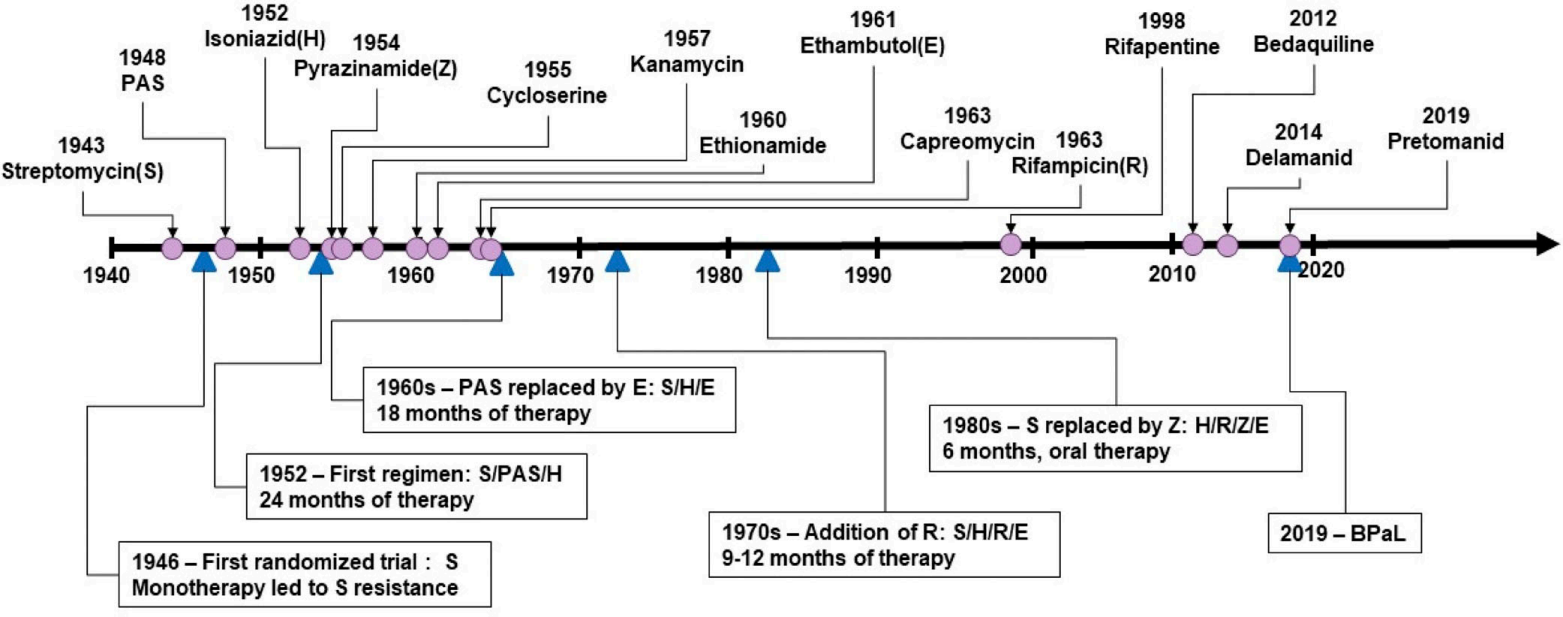
**Evolution of TB therapies.** Reprinted with permission from the TB Alliance (2020).

Much effort has been directed to the development of antibiotic drugs against TB. The first effective drug was streptomycin (Jones et al., 1944). Streptomycin, a substance exhibiting antibiotic activity against gram-positive and gram-negative bacteria, was shown to cure a ruptured TB abscess for the first time (Morgan and Bosworth, 1946). The initial results were so spectacular that they were publicized on the front pages of major newspapers around the world. Alas, within 2 yr, effectiveness waned due to the emergence of resistance. It was found necessary to add additional drugs to cure TB and diminish the probability of the emergence of resistance. Thus, TB gave rise to the first example of combination chemotherapy for any infectious disease (Fox et al., 1999; Kerantzas and Jacobs, 2017). Perhaps the most notable aspect of TB drug development is the fact that there have sadly been only four approved new molecular entities for the treatment of TB over the past 50 yr (Fig. 3; TB Alliance, 2020).

The encouraging news is that there are at least 25 drug candidates now in clinical trials (WHO, 2022a). Enormous progress has been made in shortening the course of therapy from 6 to 12 mo for drug-sensitive TB to only 4 mo (Dorman et al., 2021) or even 2 mo with an oral bedaquiline–linezolid regimen (Paton et al., 2023). Pediatric formulations now exist which save children’s lives. 1 mo of preventive therapy with isoniazid and rifapentine works very well to protect children and household contacts of TB patients, but regrettably, it reaches only a small percentage of contacts in high-burden countries (Swindells et al., 2019). Most dramatically, more effective regimens for MDR and extensively drug-resistant TB have been developed based on combinations of three new TB drugs, bedaquiline, pretomanid, and linezolid, which are remarkably effective (Conradie et al., 2022). It is broadly recognized that the shorter the duration of treatment, the greater the compliance, less resistance will develop, and ultimately transmission can be reduced. Science is doing its job; the problems of strategy and implementation remain great challenges. New and affordable drugs continue to be needed to shorten TB treatment and reduce adverse effects. Implementation of new TB regimens to reach patients in countries with a high TB burden remains essential.

Ideally, it will be important to have biomarkers for measuring the effectiveness of treatment and establishing a “cure.” Cultures remain time-consuming and insensitive (Mitchison, 1993). Chest x rays, PET-CT, and inflammatory markers commonly used to assess the response to treatment are not widely available in high-burden countries and are difficult to standardize (Walzl et al., 2011) Even after “cure,” inflammation and persistence of live *Mtb* can persist in some patients (Beltran et al., 2020). Blood transcriptional TB signatures, which are diminished on effective treatment, have been reported (Bloom et al., 2012; Cliff et al., 2013; Thompson et al., 2017). Reduced treatment response signatures have been shown to correspond with their clinical treatment response, discriminating both drug-resistant and “not cured” patients (Tabone et al., 2021; Thompson et al., 2017) and supporting the identification of patients not responding to treatment. Such an approach for determining efficacious response to treatment or not will undoubtedly be of use in assessing the efficacy of new anti-TB drugs, singularly and in combination, and therefore should facilitate and speed up the development of badly needed drugs against TB. True biomarkers to monitor TB treatment success are needed to assess the response to treatment, to determine the required treatment duration and therefore adapt drug treatment regimens, and most importantly, to accelerate testing of new anti-TB drugs under development.

## Vaccines

It is a general principle of public health that it is more cost-effective in both human and economic terms to prevent rather than treat infectious diseases and that vaccines represent the most effective tools for the prevention of infectious diseases. The story of TB vaccines has recently been superbly reviewed (Kaufmann, 2021). BCG is an attenuated live vaccine derived by 239 serial passages of a highly virulent *Mycobacterium bovis* isolate in 1908 by Calmette and Guérin. BCG was shown to protect mice, guinea pigs, cows, and NHP against *Mtb* challenge, and in 1921, it was shown to protect human subjects (Bloom and Fine, 1994). BCG was increasingly used in Europe during the 1920s until a terrible accident occurred in Lubeck, Germany, when 72 of 251 vaccinated infants died of fulminating tuberculosis (Kaufmann, 2021). It was later shown to be the result of cross-contamination with virulent *Mtb* grown in the same incubator. Nevertheless, the tragedy raised such great skepticism that no new TB vaccine underwent clinical trials for 50 yr. BCG currently remains the most widely utilized vaccine in the world, given to 104 million children around the world around the time of birth. While it may protect children against disseminated disease (Trunz et al., 2006), since children are not the major source of transmission, it has not had a great impact on the total burden of TB. Beyond TB, BCG has adjuvant effects and has been found to provide non-specific protection against leprosy, lower respiratory disease (Biering-Sorensen et al., 2017; Prentice et al., 2021), and human superficial bladder carcinoma (Reichert and Lamm, 1984).

A huge question remains: while BCG has been shown to be protective in children against TB meningitis, BCG efficacy in adults varies from 0% to 80% in human trials in different countries (Roy et al., 2014). Skepticism about BCG raised the question of whether any vaccine can protect against TB. There are two largely forgotten but crucial studies by Johannes Heimbeck with nursing students in a TB hospital in Norway in 1936 that suggest an answer (Heimbeck, 1931, 1936). He followed new nursing students for 3 yr, stratified by whether they were TST-positive upon entry and had latent infection or were naïve. The results revealed that protection in TST-positive nurses was 96% relative to TST-negative. This finding was later confirmed in a metanalysis of multiple studies of the protection of TST-positive individuals against reinfections (Andrews et al., 2012). Similarly, in 1947, Heimbach immunized TST-negative nurses with BCG and found BCG to be 50% protective (Heimbeck, 1947), a finding later confirmed by a metanalysis of 13 large trials (Mangtani et al., 2014). Virulent *Mtb* is clearly not acceptable as a vaccine because it can lead to active disease, but these historic studies reveal that it should be possible to engender high levels of protection, as in the case of infected nurses, if we could develop a safe vaccine as effective and persistent as live *Mtb*.

Two large critical trials of BCG have produced widely divergent results. A British Medical Research Council trial in 54,000 TST-negative adolescents showed that the BCG vaccine was 84% protective at 5 yr and still 77% protective at 20 yr (Hart and Sutherland, 1977). In that trial, another mycobacterial vaccine, *Mycobacterium microti*, a vole bacillus that also infects mice but is not a human pathogen, was given in a third arm of the trial, and surprisingly was as protective as BCG at both 5 and 20 yr. This inevitably raises the question of whether unique *Mtb*-specific protective antigens are essential to protection. The second trial, carried out in 260,000 people in South India over 15 yr using two BCG strains, found no protection at any age group (Bull World Health Organ, 1979). How does one reconcile these two trials? A major difference between these trials is that exposure in the UK to environmental NTM was very low. It had been shown (Palmer and Long, 1966) that infection of guinea pigs with different species of atypical mycobacteria provided varying degrees of protection against TB in guinea pigs, with some, e.g., *Mycobacterium kansasii*, fully as effective as BCG. In South India, by the age of 9, two-thirds of Indian children in the trial area were TST positive for an avian PPD, and by age 15, 97% of children were TST positive. Our hypothesis was that exposure to some NTM in the environment may provide sufficient cross-protection so as to obscure the detection of additional vaccine protection by BCG in areas with high environmental mycobacteria exposure, a potential problem for future trials (Bloom and Fine, 1994). Recent epidemiologic studies indicate that differences in exposure to *Mtb*, as well as NTM, may contribute to the differences between the trials (Mangtani et al., 2014; Martinez et al., 2022, 2023).

The challenge remains whether a safe vaccine as effective as natural *Mtb* infection can be created that will be >50% effective in all countries. While there are currently 176 trials of COVID-19 vaccines (WHO, 2023), in September 2022, there were only 16 TB vaccine candidates in clinical trials: four in Phase I, eight in Phase II, and four in Phase III (WHO, 2022a; Fig. 3). They include candidates to prevent TB infection and TB disease and candidates to help improve the outcomes of treatment for TB disease (TBVI, 2022). One is a recombinant BCG expressing listeriolysin and urease, VPM1002, designed to increase survival in phagosomes and generate cytotoxic T cells (see review by Kaufmann, 2021); a second is a subunit vaccine containing three antigens unique to *Mtb* not found in BCG (Tkachuk et al., 2020), and a third is *Mycobacterium indicus pranii* (MIP), an atypical mycobacterial vaccine which showed some efficacy in leprosy trials (Katoch et al., 2008). Two recent Phase 2 trials in adolescents in South Africa, Zambia, and Kenya give encouragement. In one case, a novel fusion protein from *Mtb* in a synthetic adjuvant (H4:IC31) was compared with BCG revaccination in a neonatally BCG-vaccinated but IGRA-negative population. While the recombinant vaccine was only 30.5% protective against infection, as measured by sustained IGRA (Quantiferon) positivity, repeat BCG as a control was surprisingly found to be 45% effective in inducing sustained positivity (Nemes et al., 2018). In the second trial, another *Mtb* fusion protein, M72, incorporated into a saponin-containing adjuvant (M72:AS01_E_) was 50% protective against disease in IGRA-positive, *Mtb*-exposed adolescents (Van Der Meeren et al., 2018) and sustained over 3 yr (Tait et al., 2019). Plans have recently been announced for a large Phase 3 trial of this vaccine in 50 sites in Africa and Asia in both IGRA-positive and -negative subjects (https://www.gatesfoundation.org/ideas/media-center/press-releases/2023/06/funding-commitment-M72-tb-vaccine-candidate). In addition, a genetically attenuated live *Mtb* strain with deletions of two genes associated with virulence (MTBVAC) is now in Phase 3 trials in Africa (Martín et al., 2021). Obviously, there is a critical need for additional sites with laboratory capacity and funding to clinically evaluate more candidate vaccines in Phase 3 efficacy trials. A review of TB vaccine trials over a half-century would suggest that an ideal vaccine should be demonstrably safe, contain multiple antigens of Mtb that can engender a long-lived cellular immune response, either in the form of a live attenuated vaccine or a subunit vaccine with an effective adjuvant.

## Take-home messages

TB is not a single entity but rather comprises a complex spectrum of disease in which immune responses are critical to both protection and pathogenesis. TB is difficult to study since the pathogen grows slowly, requires biohazard safety conditions, has limited access to lesions of TB patients, and studies of dynamics of infection and immune responses are severely constrained.

Much has been learned about the pathogen *Mtb*, its structure, genome, genetic variation, antibiotic resistance patterns, and molecular pathways. The genome sequences of thousands of isolates are now known and cataloged, and *Mtb* can now be genetically manipulated. This has enabled the development of potential new molecular diagnostics and the development of new drugs and novel vaccine candidates, many of which are still under investigation.

The general problems of antimicrobial resistance and specifically of MDR TB have spurred the recent development of new combination regimens that shorten the time of treatment (Paton et al., 2023), increasing treatment completion in patients and contacts (Sandul et al., 2017; Macaraig et al., 2018).

While no animal model faithfully represents the full spectrum of human TB, the study of animal models has been indispensable and enormously insightful. The study of granuloma function in NHP and human lung specimens has provided insight into the multiplicity of cell types involved in restricting the growth and killing of the pathogen. From many studies, it is clear that the majority of immunocompetent people infected with *Mtb* cure or restrict their infection. There are multiple stages in *Mtb* infection where the infection can be controlled. Much is known about events at later stages, where multiple cell types mobilizing in granulomas appear important in controlling the infection. What is less well understood are the protective mechanisms early in infection—for example, in the alveoli, in draining lymph nodes, and in spleen and lungs—that have the capacity to sterilize or control infection at early stages, preventing the pathology and tissue damage that are so debilitating.

New, safe vaccines more effective than BCG are being developed, but still, only three new candidates are in Phase 3 trials. Funding and identification of global sites for more rapid trials and vaccine production are badly needed to accelerate their testing and implementation.

In evaluating the scientific literature on TB, it is important to keep in mind the fundamental scientific principle that correlation does not imply causation (Popper, 2014). Over the past half-century, many immunological and molecular factors have been reported to be associated with protection in humans. This includes many different cell types—T cells, NK cells, innate lymphoid cells, macrophages and myeloid cells, mast cells, and fibroblasts associated with healing lesions and granulomas. RNAseq studies have revealed changes in a panoply of cytokines, transcription factors, and signaling molecules that are associated with protection and others with pathogenesis. Plausible hypotheses are made as to how they each may be important. But not every factor associated with lesions will actually be critical for protection. We think we know some *necessary* conditions for protection; for example, gene knockouts in mice and anti–T cell antibody experiments in NHP and human Mendelian deficiencies have established that CD4 Th1 cells and CD8 CTL and IFN-γ and TNFα appear to be involved in protection. What we critically do not know is what cell types, cytokines, and molecular mechanisms are *necessary* and *sufficient* for protection. If we did, it would be possible to establish specific biomarkers that define and predict whether latently infected individuals will control their infection, whether drugs induce true cures, and which vaccines will engender protective responses. Biomarkers of protection would profoundly accelerate the development and effective targeting of candidate vaccines and drugs and reduce the size, duration, and cost of clinical trials.
